# Profiling of ribose methylations in ribosomal RNA from diffuse large B-cell lymphoma patients for evaluation of ribosomes as drug targets

**DOI:** 10.1093/narcan/zcaa035

**Published:** 2020-12-22

**Authors:** Nicolai Krogh, Fazila Asmar, Christophe Côme, Helga Fibiger Munch-Petersen, Kirsten Grønbæk, Henrik Nielsen

**Affiliations:** Department of Cellular and Molecular Medicine, The Panum Institute, University of Copenhagen, 3B Blegdamsvej, 18.2.20, DK-2200 Copenhagen N, Denmark; Department of Hematology, Rigshospitalet, DK-2200 Copenhagen N, Denmark; Biotech Research & Innovation Centre (BRIC), University of Copenhagen, DK-2200 Copenhagen N, Denmark; Novo Nordisk Foundation Center for Stem Cell Biology, DanStem, Faculty of Health Sciences, University of Copenhagen, DK-2200 Copenhagen N, Denmark; Department of Pathology, Rigshospitalet, DK-2200 Copenhagen N, Denmark; Department of Hematology, Rigshospitalet, DK-2200 Copenhagen N, Denmark; Biotech Research & Innovation Centre (BRIC), University of Copenhagen, DK-2200 Copenhagen N, Denmark; Novo Nordisk Foundation Center for Stem Cell Biology, DanStem, Faculty of Health Sciences, University of Copenhagen, DK-2200 Copenhagen N, Denmark; Department of Cellular and Molecular Medicine, The Panum Institute, University of Copenhagen, 3B Blegdamsvej, 18.2.20, DK-2200 Copenhagen N, Denmark; Genomics group, Nord University, 8028 Bodø, Norway

## Abstract

Cancer cells are addicted to ribosome biogenesis and high levels of translation. Thus, differential inhibition of cancer cells can be achieved by targeting aspects of ribosome biogenesis or ribosome function. Using RiboMeth-seq for profiling of the ∼112 2′-O-Me sites in human ribosomal RNA, we demonstrated pronounced hypomethylation at several sites in patient-derived diffuse large B-cell lymphoma (DLBCL) cell lines with a more severe perturbation in ABC-DLBCL compared to GBC-DLBCL. We extended our analysis to tumor samples from patients and demonstrated significant changes to the ribosomal modification pattern that appeared to consist of cell growth-related as well as tumor-specific changes. Sites of hypomethylation in patient samples are discussed as potential drug targets, using as an example a site in the small subunit (SSU-C1440) located in a ribosomal substructure that can be linked to DLBCL pathogenesis.

## INTRODUCTION

Diffuse large B-cell lymphoma (DLBCL) is the most common non-Hodgkin’s lymphoma in adulthood accounting for 30–40% of all non-Hodgkin’s lymphomas (1). Treatment of DLBCL is complicated by the fact that it is a heterogeneous diagnostic category comprising different subtypes that arise by distinct pathogenetic pathways. DLBCL patients are initially treated with anthracycline-based multi-agent chemotherapy in combination with immunotherapy. Complete cure can be achieved with standard treatment leading to 5-year progression-free and overall survival rates of ∼60% and ∼65%, respectively. Disease stage and patient age as well as specific molecular aberrations, such as MYC, BCL6 and BCL2 translocations, are associated with poor prognosis. Although most DLBCL patients are cured with the standard immunochemotherapy, 10–15% of DLBCLs are primary refractory and 20–30% relapse (2,3). The main subtypes of DLBCL probably originate from B cells at different stages of differentiation, which forms the basis for the classification of DLBCLs in germinal center B-cell-like DLBCL (GBC-DLBCL), activated B-cell-like DLBCL (ABC-DLBCL) and primary mediastinal large B-cell lymphoma (PMBL) (4,5). Cells from all three subtypes have an abundance of genomic insults, but the frequencies of distinct abnormalities differ as do the transcriptional profiles relating to the main oncogenic pathways. These differences provide for new strategies of distinct subtypes based on molecular typing.

An alternative approach to cancer treatment relies on the addiction of cancers to ribosome biogenesis (6). Ribosome biogenesis consumes ∼80% of the energy in proliferating cells and differential inhibition of cancer cells can be achieved by targeting this process, e.g. by inhibition of RNA pol I by small molecule drugs that induce nucleolar stress and p53 activation leading to apoptosis (7). It has recently been demonstrated in work from our group (8) and others (9) that the ribosomes of cancer cell lines differ in ribose methylation of rRNA compared to cells in normal differentiated tissues. Ribosomal RNA is a well-known drug target in combatting infections, and in several cases, ribose methylations are implicated in antibiotic resistance (10–12). Thus, an alternative to inhibition of rRNA synthesis in cancer would be to target mature ribosomes based on differences in their rRNA modification pattern compared to the ribosomes in normal cells. Human rRNA is chemically modified at >200 residues during ribosome biogenesis (13). By far the most abundant are ribose methylations and pseudouridylations that together with a few base acetylations almost all are installed through guide RNAs that target a generic modification enzyme to specific residues. The remainder of the modifications are installed by dedicated enzymes that recognize sequence and/or structural features in the rRNA. In the case of ribose methylation, the box C/D guide RNAs recognize their targets by base pairing, allowing the methyltransferase, fibrillarin, to carry out methylation of the 2′-OH of the target residue (13). The guide RNAs are encoded within introns of host genes that can either be protein coding or encode ncRNA species. This provides for a flexible system that allows the methylation profile of the rRNA to be regulated through the expression and activation of guide RNAs. In this respect, it is interesting to note that the box C/D RNAs are among the most deregulated RNAs in cancer (14,15), and recently, a major systematic effort was made to map their expression in multiple cancers (16). We developed a sequencing-based method on the Ion Torrent platform, RiboMeth-seq (17), for profiling the ∼112 2′-O-Me sites in human rRNA and showed distinct profiles in HeLa cervical and HCT116 colon carcinoma cancer cell lines (8). Subsequently, a RiboMeth-seq protocol adapted to the Illumina sequencing platform was published (18) and used to profile the same cell lines (9,19) [for a comparison of the two protocols and other methods for profiling 2′-O-Me sites, see (20)]. Along with the rRNA profiling, RiboMeth-seq provides low-coverage RNA-seq of the guide RNAs that has proven to be good estimates of their expression levels.

Here, we present RiboMeth-seq profiling of four cell line models of DLBCL as well as of samples from 17 primary patient DLBCLs. The cell lines comprised models of GBC-DLBCL (RL and HT) and ABC-DLCBL (OCI-Ly3 and U-2932). The patient DLBCL samples represented a wide range of clinical manifestations and were compared to reactive lymph nodes from three donors. We observed specific changes in ribose methylation pattern in DLBCL samples that seems to correlate with proliferation, and we suggest that such changes are a molecular feature of DLBCL. To our knowledge, this is the first demonstration of rRNA methylation changes in cancer specimens from patients and the observations are discussed in the context of targeting the changes in cancer treatment.

## MATERIALS AND METHODS

### Cell culture

Human lymphoma cell lines were maintained in RPMI-1640 medium (Gibco) supplemented with final concentrations of 25 mM HEPES (Gibco), 1 mM sodium pyruvate (Gibco), 100 U/ml penicillin–streptomycin (Gibco) and heat-inactivated fetal bovine serum (Gibco) at 10% for RL, HT and U-2932 or 20% for OCI-Ly3.

### Patient samples

Nineteen fresh-frozen DLBCL biopsies from patients diagnosed with primary DLBCL were obtained from the Department of Pathology at our institution (University of Copenhagen, Rigshospitalet). The specimens were collected during 2002–2013. The diagnoses were based on standard histology and immunophenotyping according to the WHO classification. All tissue samples were fresh frozen, stored at −80°C and embedded in Tissue-Tek O.C.T. Compound prior to RNA extraction. All biopsies had a tumor percentage of >80%. The majority of samples have previously been classified according to Hans’ classification into GCB or non-GCB subtypes (21). Reactive enlarged non-malignant lymph nodes (RLNs) were used as controls and obtained from six individuals. The project is approved by the National Ethics Committee (2002446) and by the Data Protection Committee according to the Danish ethical regulations (RH-2016-170).

### Ki-67 staining

Sections of formalin-fixed, paraffin-embedded samples of DLBCL (3 μm thick) were used for immunohistochemical evaluation of Ki-67 antigen, using the Ki-67 antibody MIB-1 clone (DAKO/Agilent GA626) following the manufacturer’s instructions. The staining was performed on Dako Omnis (Agilent), utilizing the EnVision Flex+ detection kit (GV800). The primary antibody was diluted using Antibody Diluent (Dako DM830) and subsequently incubated for 20 min. The sections were counterstained with hematoxylin and the percentage of stained Ki-67 nuclei was evaluated [Ki-67 labeling index (Ki-67LI)].

### Isolation of whole-cell RNA from cells, solid tissues, tumor biopsies and reactive lymph nodes

Whole-cell RNA was isolated from cell cultures using Trizol Reagent (Thermo Scientific) according to the manufacturer. RNA from tumor biopsies and benign RLNs was isolated using Trizol Reagent and Qiagen miRNeasy kit according to the manufacturer. Whole-cell RNA from solid tissues pooled from five adult donors was purchased from BioChain [catalog numbers R1234035-P (brain), R1234171-P (skeletal muscle) and R1234149-P (liver)].

### RiboMeth-seq analysis

Five micrograms of whole-cell RNA was degraded by alkaline hydrolysis, size fractionated and ligated to adaptors as previously described (17,22). In brief, a 20–40-nucleotide fraction of alkaline degraded RNA was excised and purified from gels. Adaptors were ligated to the RNA using a tRNA ligase and cDNA was synthesized using SuperScript IV reverse transcriptase (Thermo Scientific). Final libraries were sequenced on an Ion Proton sequencer, and reads were mapped against human rDNA (8) and SNORD sequences using Bowtie2. RiboMeth-seq (RMS) scores representing ‘fraction methylated’ were calculated as described previously (‘score C’) in (17). In a few cases, a barcode correction was applied when calculating the score (23). Misincorporation was analyzed using SAMtools and SNORD expression (RPKM) was calculated using an in-house Python script. Analyses of cell cultures were conducted in biological triplicates, whereas those of solid tissues, RLNs, and tumor biopsies were conducted in technical triplicates. RLNs and tumor biopsies used for RiboMeth-seq analysis are listed in Supplementary Table S1 in anonymized form.

### RT-qPCR analysis

Approximately 50 ng whole-cell RNA was treated with 2 U of TURBO™ DNase (Thermo Scientific) at 37°C for 20 min, extracted once with phenol:chloroform:isoamyl alcohol (25:24:1) and once with chloroform, and subsequently precipitated with 3× volume of 96% ethanol in the presence of 300 mM NaAc and 15 μg of glycogen. The DNase-treated RNA was reverse transcribed using iScript-cDNA kit (Bio-Rad) according to the manufacturer’s instructions except that the RNA was denatured in the reaction buffer at 90°C for 30 s prior to adding the reverse transcriptase. One-twentieth of the cDNA was used for qPCR analysis on a LightCycler Nano System (Roche) using FastStart Essential DNA Green Master (Roche) according to the manufacturer’s instructions. Relative fold expression was calculated using the ΔΔCq method, normalized to snRNA U6, and expression in RLN set to 1. RLNs and tumor biopsies, and DNA primers used for RT-qPCR analyses are listed in Supplementary Tables S1 and S2, respectively.

### Statistical methods

Statistical analyses were performed using Microsoft Excel and GraphPad Prism 7. RiboMeth-seq results (RMS score, fraction methylated) were expressed as mean ± standard deviation, and SNORD expression data from RiboMeth-seq (RPKM) and RT-qPCR (ΔΔCq method) as mean ± standard error of the mean. Misincorporation at base modifications was expressed as 95% confidence interval of the median. Correlations between ribose methylation sites or summed ΔRMS scores compared to Ki-67LI were analyzed using Spearman’s rank correlation. Comparison of two groups was analyzed by two-tailed Student’s unpaired *t*-test and statistically significant differences between groups were indicated as *P < 0.05, **P < 0.01 and ***P < 0.001.

## RESULTS

### DLBCL cell lines are hypomethylated at distinct sites in rRNA

**Figure 1.**
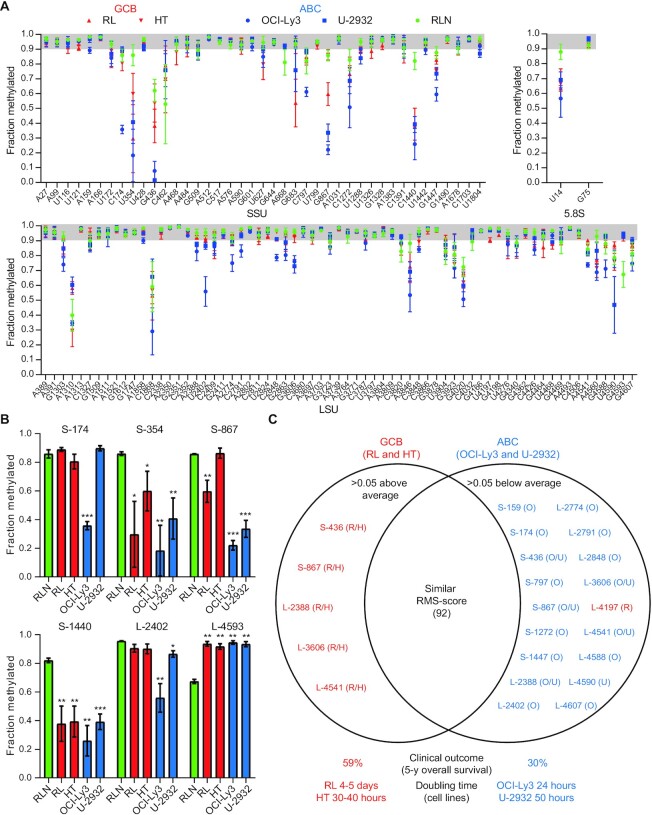
Profiling of ribose methylations in rRNA from DLBCL cell lines. (**A**) Fraction methylated at sites in the small (SSU) and large (LSU) ribosomal subunit RNA in GBC-DLBCL (RL and HT) and ABC-DLBCL (OCI-Ly3 and U-2932) as well as in RLN. The gray-shaded area represents full or close to full methylation. (**B**) Histograms comparing the methylation scores at selected positions discussed in the text. Asterisks indicate statistical significance between RLN and the cell line in question. (**C**) Diagram depicting deviations from average methylation scores in GBC- and ABC-DLBCL. The sites that deviate the most from the average RMS score (fraction methylated) from all cell lines are highlighted and sites scoring statistically significant above average are to the left and sites scoring below average to the right. The deviating sites in the GCB set (in red) and the ABC set (blue) cluster, with the score for SSU-L4197 from the RL cell line as an outlier that scores below average in contrast to other deviating sites from the GCB set. R: RL; H: HT; O: OCI-Ly3; U: U-2932.

In order to define a baseline for perturbations of rRNA modifications in DLBCL, we first profiled ribose methylation levels by RiboMeth-seq in two cell lines representing GCB-DLBCL (RL and HT) and two cell lines representing ABC-DLCBL (OCI-Ly3 and U-2932). As a reference, we profiled three reactive lymph nodes (Figure 1A and Supplementary Data S1). In the reactive lymph nodes, the large majority of sites were fully or close to fully methylated. In contrast, hypomethylation was observed in all four cell lines. Importantly, this was not due to a general low methylation level, but affected a small subset of sites only. Similar observations have been made previously by us (8,23) and others (9,19) with cervical (HeLa), colon (HCT116) and T-cell (Jurkat) cancer cell lines. Most hypomethylated sites were shared with other cancer cell lines, with a few sites that may be distinctive of DLBCL (e.g. SSU-G683), although this must await a more comprehensive analysis of cancer cell lines.

The GBC- and ABC-DLBCL cell lines showed some common characteristics; e.g. SSU-C1440 was hypomethylated in all cell lines compared to RLN, and conversely, LSU-G4593 was hypermethylated compared to RLN (Figure 1B). To compare cell lines, we calculated the deviation of the RMS scores in each cell line from the average RMS score of all cell lines (Figure 1C). Importantly, the ABC lines that represented the more aggressive lymphoma subtype clustered and had many more hypomethylated sites and a larger extent of hypomethylation at several shared sites (e.g. SSU-G867; Figure 1B) compared to GCB lines. Among the two ABC lines, OCI-Ly3 was consistently the most hypomethylated. This cell line had the shortest doubling time (24 h) of the four cell lines analyzed. We conclude that patient-derived cell lines are hypomethylated at a subset of rRNA methylation sites, and that ABC lines are affected at more sites and to a higher degree.

### rRNA modifications in DLBCL patient cells differ from normal cells

**Figure 2.**
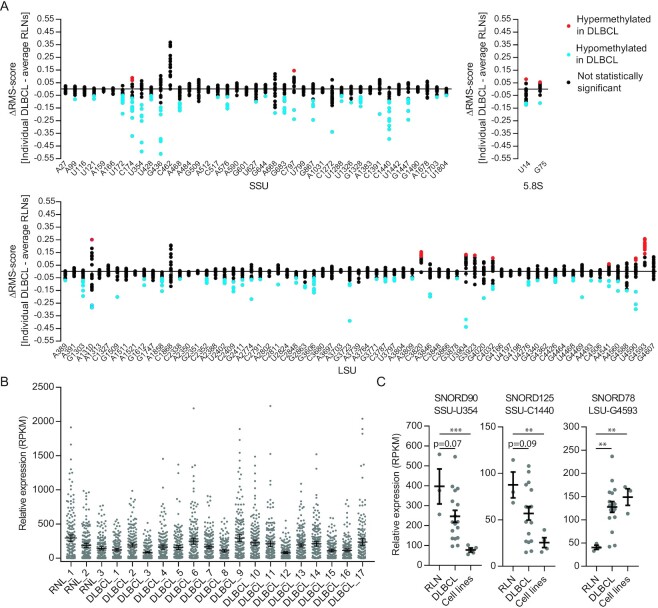
Profiling of ribose methylations and guide RNAs in patient tumor samples. (**A**) Summary of RiboMeth-seq analysis depicted deviations from the average of the three RLN control samples. Statistically significant (*n* = 3) data points are labeled in blue and red, representing hyper- and hypomethylation, respectively. SSU: small ribosomal subunit; LSU: large ribosomal subunit. (**B**) Normalized expression levels of all rRNA targeting box C/D snoRNAs in DLBCL tumor samples and RLN controls. (**C**) Expression levels of three box C/D snoRNAs that varied in expression levels in parallel to changes in rRNA methylation levels. Bars represent mean values.

Patient-derived cancer cell lines adapt to propagation in the laboratory and may accumulate additional mutations. Thus, we profiled patient DLBCL samples to validate that rRNA hypomethylation was of clinical relevance to DLBCL. Our sample comprised 17 DLBCLs, of which 7 were typed as GCB, 4 as non-GCB and 6 were not typed (Supplementary Table S1). The RMS scores are listed in Supplementary Data S1, and the profiles are shown in Supplementary Figure S1. In order to highlight the differences between tumor and normal tissue, the results are presented as deviations of the scores from the average scores of three RLN samples in Figure 2A. As with cell lines, sites were affected in a non-random fashion. Most sites were unaffected and the affected sites comprised a few cases of hypermethylations and several cases of hypomethylations. At most sites, only some of the tumor samples showed statistically significant deviations; however, at a few sites, almost all (SSU-U354 and SSU-C1440) or all samples (LSU-G4593) deviated from the control. Interestingly, the sites in the small subunit, in particular those located in the 5′ domain, were much more hypomethylated than the sites in the large subunit. This may provide clues to the functional significance of the changes as the small subunit is specifically implicated in mRNA recruitment and scanning. Information on the most affected sites is provided in Table 1 and the positions of the sites in the ribosome structure (24,25) are shown in Supplementary Figure S2.

**Table 1.** List of the 2′-O-Me sites most affected in DLBCL

	Pos	h/H	Dom	*E. coli* #	M	Z	Y	SNORD		Host gene	RMS (RLN)	Comment
S	U172	h8	5′	173	+	+	−	45A	D′	RABGGTB	94	C
								45B	D′	RABGGTB		
	C174	h8	5′	175	+	−	−	45C	D′	RABGGTB	86	G
¤	U354	h7	5′	G237?	+	−	−	90*		RC3H2	86	G
	G436	h13	5′	C316?	+	+	−	100	D′	RPS12	62	G
	A468	h14	5′	G348?	+	+	+	83A	D	RPL3	94	C
								83B		RPL3		
	A576	h17	5′	U467?	+	−	−	93	D	SNHG26	93	?
¤	G683	J20/21	5′/C	G587?	+	−	−	19	D	GNL3	94	C
								19B	D	GNL3		
¤	G867	ES6	C	−	+	−	−	98	D′	CCAR1	86	G
	G1328	h34	3′M	C1051	+	+	+	32A	D	RPL13A	96	C
	C1440	h39	3′M	−	−	−	−	125*	D	AP1B1	82	C
	G1447	h38	3′M	A1152?	+	−	−	127	D	PRPF39	88	G
L	G1303	H26	II?	C565?	+	+	−	21	D	RPL5	93	G
	A1858	H39	II	U963?	+	+	+	38A	D′	RPS8	99	G
								38B	D′	RPS8		
	G3606	H64	IV	U1765?	+	+	−				96	(G)
	G4593	H96	VI	2679U?	+	+	−	78*	D′	GAS5	67	G

The nucleotide position in the small (S) or large ribosomal subunit RNA is listed together with information of the structural domain (Dom) and the helix (h for small subunit and H for large subunit helices, respectively) or junction (J) element. The corresponding nucleotide position in *Escherichia coli* rRNA was inferred from a structural alignment. The conservation of the 2′-O-Me site was based on RiboMeth-seq analyses in the mouse (M) (27), zebrafish (Z) (28) and yeast (Y) (17). The assignment of the SNORD responsible for guiding the methylation, the associated D-box element and the host gene was from (8,25), and the RMS score was from RLNs as reported in the present paper. Based on a comparison of tumor samples and samples from mouse development, 2′-O-Me sites were tentatively assigned as generally growth-related (G) or specifically related to cancer (C). *: SNORD level appears limiting for methylation level; ¤: in close proximity in structure.

We next asked whether the methylation changes occurred in a coordinated fashion when individual tumors were compared. This would require extensive coordination because the box C/D guide RNAs are expressed from many different host genes. Indeed, when all RiboMeth-seq profiles were superimposed, a somewhat erratic picture emerged, suggesting absence of a regulatory pathway driving the methylation changes. However, in pairwise comparisons using Spearman’s rank correlation, it appeared that there was a coordinate decrease in methylation levels at three of the most affected sites (Supplementary Figure S3). Increased methylation at LSU-G4593 seemed to anticorrelate with hypomodification at other key sites, but this fell short of the statistical test. The coordinate changes were reminiscent of what was observed in the comparison of GCB and ABC cell lines (Figure 1B) and may reflect a drive toward a distinct ribosomal methylation signature in the tumors. When methylation profiles of cell lines (Figure 1A) and patient DLBCLs (Supplementary Figure S1 and Figure 2A) were compared, a significant overlap in terms of both sites affected and the extent of hypo- or hypermethylation was observed. Thus, the cell lines appeared to be relevant models of the methylation changes in primary DLBCL specimens.

RiboMeth-seq includes data on misincorporation during cDNA synthesis caused by modifications on the WC face of the bases that can be used as a proxy for modification levels. Here, we found SSU-m^1^acp^3^Ψ1248, SSU-m^7^G1639 and SSU-m^6^_2_A1851 to be hypomodified in both cell lines and primary DLBCL, in contrast to LSU-m^1^A1309 and LSU-m^3^U4500 that were unaltered (Supplementary Figure S4 and Supplementary Table S3). SSU-m^1^acp^3^Ψ1248 was recently found to be hypomodified in many cancer types (26). Thus, hypomodification in rRNA appears as a widespread phenomenon in cancer.

### Key box C/D snoRNA guides are deregulated in DLBCL

In order to understand the non-random distribution of affected sites overall as well as in individual DLBCL, we experimentally addressed the expression levels in the lymphomas of the box C/D snoRNAs that guide the methylations of rRNA. RiboMeth-seq is performed on whole-cell RNA and thus provides a low-coverage RNA-seq for small RNAs at intermediate abundance, such as the box C/D snoRNAs. This approach has the advantage that partial alkaline degradation of the RNA prior to adaptor ligation leaves many ends representing each RNA species, thus reducing bias in the adapter ligation step. The overall levels of box C/D snoRNAs with rRNA targets differed somewhat among the tumor samples, but were within the range of the RLN controls (Figure 2B). Many snoRNAs showed significant variation in expression, but no overall correlation between snoRNA level and the stoichiometry of ribose methylation was observed. Three of the most affected sites are guided by box C/D snoRNAs that were expressed at relatively low levels, and in these cases, the methylation stoichiometries were paralleled by the snoRNA levels. SSU-U354 was hypomethylated in DLCBL cell lines and the guide, SNORD90, was expressed at much lower levels in the cell lines compared to RLNs (Figure 2C). The DLBCL tumor samples differed considerably in SNORD90 expression as well as in methylation at SSU-U354, but the mean value was in between those of cell lines and RLNs as were the methylation scores. A parallel example was found at SSU-C1440 guided by SNORD125 (Figure 2C). At LSU-G4593, the methylation score was high in the cell lines and lower in the RLNs and this was paralleled by expression levels of SNORD78. Again, the DLBCL samples displayed considerable variation in SNORD expression (Figure 2C), but in this case the methylation scores were consistently elevated compared to the control. SNORD78 is encoded within the ncRNA Gas5 host gene and in the mouse its expression is uncoupled from the expression from other guide RNAs expressed from the Gas5 transcript through alternative splicing and NMD (27). The differences in SNORD expression between RLN, on the one hand, and DLBCL cell lines and patient samples, on the other hand, revealed by low-coverage RNA-seq were confirmed by RT-qPCR analyses (Supplementary Figure S5). Thus, it appears that some key rRNA methylations are regulated through expression levels of the corresponding guide RNAs.

### Many methylation changes appear to reflect cellular growth

**Figure 3.**
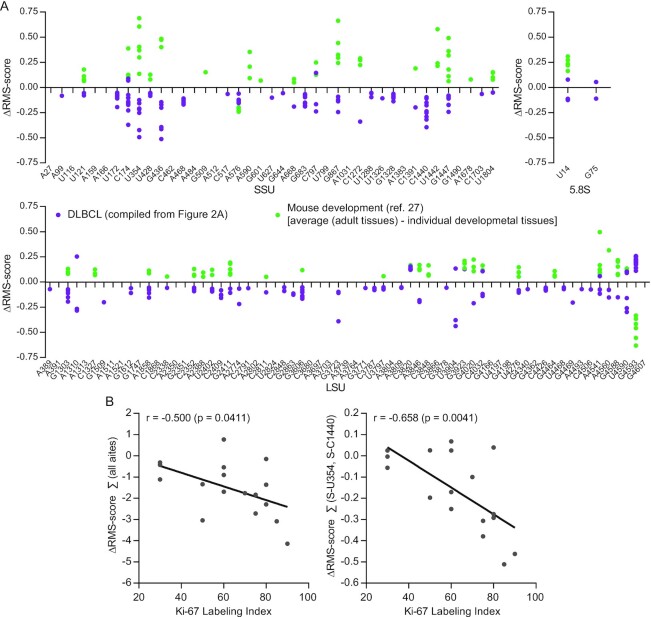
(**A**) Comparison of rRNA methylation changes (ΔRMS score) during development and in DLBCL. Purple dots represent statistically significant methylation changes taken from Figure 2A (i.e. combining red and blue dots) and were calculated by subtracting values in RLNs from values obtained from individual DLBCL tumors. Green dots represent statistically significant differences in mouse development from (27) calculated by subtracting values from embryonic tissues from values of the matching adult tissues. (**B**) Correlation of the sum of ΔRMS scores at all sites (left) or of SSU-U354 and SSU-C1440 only (right) and the Ki-67LI for all patient samples.

Cancer cells are addicted to ribosome biogenesis in order to support rapid growth (6). To distinguish growth-related changes from cancer-specific changes in ribosome methylations, we compared the changes in DLBCL depicted in Figure 2A with changes observed during development from our recent study of mouse development (27). The mouse study focused on comparison of five tissues from E16.5 embryos and matching adult tissues and found an increase in methylation levels at a subset of positions as well as a decrease in methylation at a few sites. Samples that showed statistically significant changes in the mouse study were compared to the ΔRMS scores from the present analysis of DLBCL samples presented in Figure 2A. With the exception of SSU-C1440 and LSU-A1310 that have no guide RNAs in the mouse and thus are not methylated, the inventory of rRNA ribose methylation sites appears similar in the two organisms, although a few additional sites may be found in specialized cells or tissues in the future. The idea of this comparison, plotted in Figure 3A, was to compare two pattern changes, i.e. to depict which sites display hypo- or hypermethylation and to what extent they vary within the set. Thus, the absolute changes in the two datasets are not compared as they are compared against different controls of differentiated tissues. The most striking feature in the comparison was the many sites that showed both an increase in methylation during development and a decrease in methylation in cancer. We propose that these changes reflect differences in the proportion of dividing cells to resting cells in the sample. In addition to the supposedly growth-related site changes, an equal number of sites were hypomethylated specifically in DLBCL. Around one-third of sites were specifically hypomethylated in one or more developing mouse tissues. More sites in SSU than in LSU showed changes and affected sites clustered in the ribosome 5′ domain of the SSU and domains DII and DVI of the LSU (Table 1 and Supplementary Figure S2). To test the idea that the methylation changes reflected cellular growth, we measured the Ki-67LI, a marker for proliferation. As shown in Figure 3B, the sum of all methylation changes correlated with the Ki-67LI and this correlation was largely driven by changes at two (SSU-U354 and SSU-C1440) of the three positions for which methylation scores correlated with expression of the SNORD RNA guides. From this analysis, we propose that the rRNA methylation in DLBCL can be roughly divided into sites that are related to rapid growth and sites that may be DLBCL specific. The former comprised the sites that change the most. The latter group comprised sites that were affected in fewer tumors and may be related to genomic insults that vary among the tumors.

## DISCUSSION

Using RiboMeth-seq, we demonstrated methylation changes, predominantly hypomethylation, at a subset of the ∼112 2′-O-Me sites in rRNA from cell lines and primary DLBCL samples. We and others have previously demonstrated hypomethylation in HeLa and HCT116 cells (8,19) and methylation changes in developing tissues of the mouse (27) and zebrafish (28). In contrast, RNA extracted from tissues that largely is composed of differentiated cells showed close to full methylation at the vast majority of sites as demonstrated by the profiles of RLNs and of several adult tissues, e.g. liver, skeletal muscle and brain (Supplementary Figure S6). Only SSU-C1440 was detected as a new site in these analyses, suggesting that the current list is close to being the complete set of 2′-O-Me sites in human rRNA. Importantly, the subset of sites affected as well as the extent of hypomethylation in various settings appeared informative; e.g. cultured cells of cancerous origin displayed high variability and sometimes profound perturbations of the methylation patterns compared to more distinct changes in biological settings, e.g. during development (27,28). In the present case, the ABC lines representing the subtype of DLBCL with the poorest prognosis had a considerably more perturbed methylation pattern than the GCB lines, and among the two ABC lines, OCI-Ly3, which is the most proliferative cell line, was the most affected (Figure 1). Thus, it appears that the cell lines reflected aspects of DLBCL with respect to rRNA modification patterns. The primary DLBCL samples showed methylation changes at essentially the same sites as the cell lines (compare Figures 1A and 2A, and Supplementary Figure S1), albeit to a lesser extent, as expected. DLBCLs comprise a high degree of both intra- and intertumoral heterogeneity (29). To reduce contamination of wild-type RNA originating from reactive cells and connective tissue, we have selected DLBCL samples with at least 80% tumor involvement, which may have contributed to the significant overlap of ribose methylation changes observed in the DLBCL patient samples and the DLBCL cell lines that comprise a more homogeneous cell population. Importantly, Ki-67LI correlated with the overall pattern change, and specifically with methylation changes at key sites SSU-U354 and SSU-C1440, suggesting that methylation changes are related to tumor growth (Figure 3B). All DLBCL patient samples were classified as the DLBCL NOS group according to the WHO classification and obtained from treatment-naïve patients. However, the degree of methylation changes varied considerably among tumors, which may attribute to the intertumoral heterogeneity observed in DLBCL. The GCB-DLBCLs express genes characteristic of normal germinal center B cells such as CD10 and BCL6, and have ongoing somatic hypermutations (SHMs) of the immunoglobulin V genes, while the ABC-DLBCLs express genes characteristic of *in vitro* activated B cells, and do not express germinal center B cell-restricted genes or have ongoing SHMs. The ABC-DLBCLs are most similar to post-GC immunoblasts, and particularly, the NF-κB target genes are expressed in ABC-DLBCL. The ABC-DLBCL is associated with an inferior overall survival compared to GCB-DLBCL and PMBL, which is associated with a favorable overall survival relative to the other two subgroups. However, there is still clinical heterogeneity within these groups that cannot be explained by the current classification. There are a variety of genetic and epigenetic aberrations associated with the pathogenesis of DLBCL, including aberrations that are specifically associated with the cell-of-origin defined subgroups of DLBCL. Due to the limited sample size as well as differences in treatment regimen, correlation of methylation pattern and clinical data was not attempted.

Ribose methylations are guided by box C/D guide RNAs that are encoded within introns of host genes. Thus, changes in methylation pattern could be driven by changes in host gene expression and/or processing of individual box C/D guide RNAs. To analyze such contributions in DLBCL, we first inspected lists of the most frequently (>5%) mutated genes in two whole-exome sequencing studies (30,31). The studies listed 60 genes from 1001 patients and 98 genes from 304 patients, respectively. None of the genes were host genes encoding box C/D snoRNAs targeting rRNA. Transcriptomics data were not inspected because intronic encoded guide RNA expression can be uncoupled from expression of exonic products through alternative splicing and NMD (32) as indeed appeared to be the case for mouse SNORD78 (27). Instead, we experimentally addressed the expression levels of the guide RNAs themselves (Figure 2B and C, and Supplementary Figure S5). The guide RNAs are among the most deregulated transcripts in cancer (14,15) and some were demonstrated to have non-ribosomal functions of relevance to cancer (33). In the present study, we did not observe vast deregulation of the guide RNAs in DLBCL compared to RLNs (Figure 2B). We confirmed our previous observation (8) of a lack of overall correlation between the expression levels of individual guide RNAs and methylation levels at their target sites. However, for three of the most affected sites, the expression levels of the guide RNAs varied in parallel with the methylation stoichiometry. Furthermore, these were among the few sites for which we found concerted changes in the tumors. This could indicate an adaptive response at the level of ribosome modification during tumorigenesis. SNORD78 is particularly interesting as it has previously been implicated in several cancers (14,34–37), albeit without being linked to its ribosomal target. Based on its consistent upregulation in cell lines and tumors and the direct consequences in elevation of methylation levels at LSU-G4593, we suggest that this snoRNA is re-evaluated based on its function in installment of one of the key ribose methylations in rRNA.

The majority of methylation changes in DLBCL mirrored the changes observed during mouse development. This adds to the idea of relatedness between development and tumorigenesis, e.g. as observed in comparisons between developmental and pathological epithelial–mesenchymal transition (EMT) (38,39). We envisage that changes in methylation pattern on the small ribosomal subunit can impact translation through mRNA recruitment. Interestingly, increased translation from IRES-driven mRNAs due to inhibition of cap-dependent translation by the mRNA binding protein YB1 has been suggested as an inducer of EMT and enhancer of metastatic capacity (39). Alternatively, modifications may impact the trade-off between speed and fidelity of translation. Ribosome load can impact the cellular proteome and drive cancer development and progression (40). Similarly, changes in speed and fidelity of translation will impact the proteome through its impact on co-translational protein folding (41,42). It will be of interest to see whether characterization of additional cancers by RiboMeth-seq will lead to a definition of cancer ribosomes, which will constitute an additional hallmark of cancer (43) necessitated by the addiction of cancer cells to increased ribosome biogenesis and protein synthesis.

**Figure 4.**
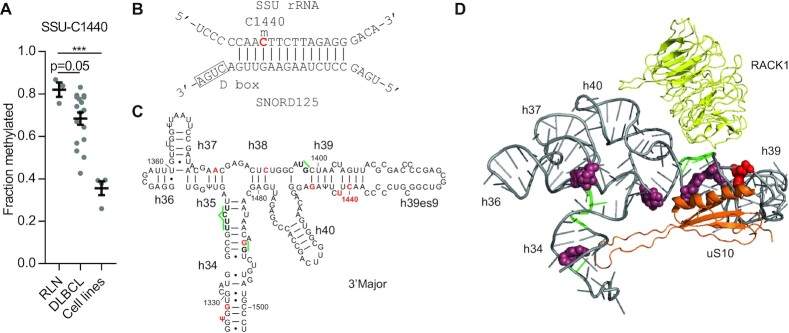
Structural context of SSU-C1440. (**A**) RMS scores at SSU-C1440 reported in this study. Bars represent mean values. (**B**) Proposed base pairing between SNORD125 and its ribosomal target. The methylated nucleotide is in red color. (**C**) Helix diagram of human rRNA helices 34–40 with ribose methylations in red color and indication in green color of nucleotides protected by eIF4B in hydroxyl radical footprinting experiments in yeast from (47). (**D**) 3D structure of the same region taken from the cryo-EM structure (4UG0) in (24) and made in PyMol. Ribose methylations are in raspberry except for SSU-C1440 that is in red, and eIF4B protected nucleotides in green. Proteins uS10 and RACK1 are in orange and yellow, respectively. The suggested base pairings in the 2D and 3D representations differ at some sites.

Ribose methylations are found at conserved and functionally important parts of the ribosome (44). One of the shortcomings of the present study and the field in general is the lack of functional studies ascribing functions to individual methylation sites, although such studies are beginning to emerge (42). As an example on how methylation changes may impact ribosome structure and function, we inspected structure models of the immediate surroundings of SSU-C1440 located in the highly irregular h39–h39ES9 that appears ideal for drug targeting because it contains unique structural features and is solvent accessible. This site was not methylated in qMS-based studies of ribosomes in human lymphoblast TK6 and HeLa cells (45), and in previous RiboMeth-seq analyses of cultured cells, the methylation score was at background levels (8,46), which was also the case for the DLBCL cell lines reported here (Figure 4A). In contrast, we report that by far the majority of ribosomes are methylated at this position in RLNs (Figure 4A), and in adult tissues (Supplementary Figure S6). The methylation is guided by SNORD125 (Figure 4B), the single guide encoded within the AP1B1 gene. The expression levels of SNORD125 (Figure 2C) and the methylation levels at SSU-C1440 (Figure 4A) varied considerably in the DLBCLs. The site is located in h39 in the h34–h40 part of the 3′ major domain of the small subunit rRNA (Figure 4C). The basal h34 is located at the top of the A site and neck of the small subunit and is conserved in sequence and with respect to three 2′-O-Me sites between yeast and humans. In contrast, h35–h40 has large patches with multiple sequence differences between yeast and humans and contains 2′-O-Me sites in humans only. The overall 3D structure is mostly conserved, suggesting that the multiple methylations play a role in conformational flexibility. Interestingly, parts of h34–h40 were protected by yeast translation initiation factor eIF4B in hydroxyl radical footprinting experiments (Figure 4C) (47) at three locations, including a site close to SSU-C1440 (Figure 4C and D). Of particular interest, overexpression of eIF4B has been implicated in the pathogenesis of DLBCL (48). Cryo-electron microscopy (cryo-EM) analyses of the human 48S pre-initiation complex involved in cap-dependent translation suggest that eIF4B stretches along the 40S subunit from the entrance to exit of the mRNA channel (49). eIF4B is required for efficient mRNA recruitment to the translation initiation complex and interacts with the helicase eIF4A that unwinds structured 5′ UTR regions during the scanning process. Additionally, eIF3d contacts h40. eIF3d has cap-binding activity and binds specifically to mRNAs with highly structured 5′ UTRs, such as *c-jun* mRNA (50). RACK1, which has been implicated in control of IRES-containing mRNAs (51), is flanking h39 opposite to uS10 (Figure 4D). RACK1 and eIF4B are both downstream effectors of intracellular signaling. eIF4B is one of the targets of PI3K–mTOR–S6K signaling and its phosphorylation by S6K or protein kinase B leads to increased protein synthesis (52). Thus, we suggest that the methylation status of SSU-C1440 differentiates ribosomes by altering the affinity for translation factors involved in mRNA recruitment. Specifically, synthesis of a large pool of ribosomes that remain unmethylated at SSU-C1440 may allow increased levels of eIF4B in DLBCL to bind more ribosomes and favor translation of mRNAs with highly structured 5′ UTRs such as DAXX, BCL2 and ERCC5 that are involved in tumorigenesis and chemoresistance (48). In this way, the ribose methylation is important in coupling cellular signaling to differential translation of the mRNA pool, and thus in shaping the cellular proteome. As the methylation status at SSU-C1440 appears to correlate with tumor growth in DLBCL and binding of nearby proteins is linked to the pathogenesis, we suggest that small molecule drugs binding to ribosomes depending on the presence of the methyl group can perturb the ribosome population in the cell in ways that may inhibit tumor growth.

In conclusion, our study supports the idea that certain box C/D guide RNAs are relevant as biomarkers in cancer, and that these guide RNAs as well as the modification they install should be considered as a novel type of cancer targets.

## DATA AVAILABILITY

RiboMeth-seq data are deposited at NCBI Gene Expression Omnibus: GSE153502.

## Supplementary Material

zcaa035_Supplemental_Files
