# Supplementary material for: Profiling of ribose methylations in ribosomal RNA from diffuse large B-cell lymphoma patients for evaluation of ribosomes as drug targets
Source: NAR Cancer. 2020 Dec 22;2(4):zcaa035. doi: 10.1093/narcan/zcaa035 (PMC8210301; doi:10.1093/narcan/zcaa035)
Supplement: zcaa035_Supplemental_Files [file zcaa035_supplemental_files.zip › Supplementary.pdf]

Supplementary for

Profiling of ribose methylations in ribosomal RNA from diffuse large B-cell lymphoma patients for evaluation of ribosomes as drug targets

Nicolai Krogh, Fazila Asmar, Christophe Côme, Helga Fibiger Munch-Petersen, Kirsten Grønbæk, and Henrik Nielsen

The Supplemental Material contains:

Data S1
Table S1
Table S2
Table S3
Figure S1
Figure S2
Figure S3
Figure S4
Figure S5
Figure S6

Data S1. Read-end numbers recorded from RiboMeth-seq library sequencings and RiboMeth-seq (RMS)-scores calculated for patient-derived cell lines, patient samples, RLNs, and normal differentiated tissues. Available online at NAR Cancer and at NCBI GEO under accession number GSE153502.

Table S1. Patient samples.

Patient number	Classification	RiboMeth-seq	RT-qPCR
DLBCL_1	GCB	X	X
DLBCL_2	Non-GCB	X	
DLBCL_3	NA	X	X
DLBCL_4	Non-GCB	X	X
DLBCL_5	Non-GCB	X	X
DLBCL_6	NA	X	X
DLBCL_7	Non-GCB	X	X
DLBCL_8	GCB	X	X
DLBCL_9	GCB	X	X
DLBCL_10	Non-GCB	X	X
DLBCL_11	GCB	X	X
DLBCL_12	NA	X	
DLBCL_13	NA	X	X
DLBCL_14	GCB	X	X
DLBCL_15	GCB	X	X
DLBCL_16	NA	X	
DLBCL_17	GCB	X	X
DLBCL_18	Non-GCB		X
DLBCL_19	Non-GCB		X
RLN_1	Benign	X	X
RLN_2	Benign	X	X
RLN_3	Benign	X	X
RLN_4	Benign		X
RLN_5	Benign		X
RLN_6	Benign		X

NA: not applicable.

Table S2. DNA primers used for RT-qPCR.

Target	Sequence
SNORD78 forward	GTGTAATGATGTTGATCAAATGTCTG
SNORD78 reverse	TTCTTCAGTGTTACCTTTGTCTAC
SNORD90 forward	GTCTAATGATGAATTTCATAGGGCA
SNORD90 reverse	GTCTTCAGATTCCACAGTAGGAG
SNORD125 forward	AGCCCCTCCTGATGATTC
SNORD125 reverse	TTCAGTCAACTTCTTAGAGGCTC
snRNA U6 forward	CTCGCTTCGGCAGCACA
snRNA U6 reverse	AACGCTTCACGAATTTGCGT

Table S3. Analysis of mis-incorporation in cDNA synthesis at base modifications on the WC-face of the nucleotide.

**SSU**												
Modification*	Nucl.		Misincorporation								Total	
$m^1acp^3\Psi$-1248	U1248	Sample:	G%	SD	A%	SD	T%	SD	C%	SD	Avg	SD
		DLBCL	0.68	0.10	6.53	2.28	-	-	72.87	1.38	80.07	2.37
		RLN	0.57	0.02	11.22	0.66	-	-	73.20	0.21	84.99	0.48
		Cell lines	0.57	0.03	10.34	0.43	-	-	69.98	1.73	80.89	2.05
m^7G-1639	G1639	Sample:										
		DLBCL	-	-	8.10	1.61	0.37	0.13	0.06	0.05	8.53	1.57
		RLN	-	-	10.20	0.57	0.50	0.11	0.06	0.02	10.75	0.69
		Cell lines	-	-	9.07	0.59	0.43	0.05	0.06	0.04	9.56	0.62
$m^6{}_2A$-1851	A1851	Sample:										
		DLBCL	0.41	0.24	-	-	1.36	0.97	-	-	1.77	1.20
		RLN	0.68	0.03	-	-	2.71	0.12	0.01	-	3.39	0.14
		Cell lines	0.50	0.04	-	-	1.84	0.15	-	-	2.34	0.18
**LSU**												
Modification	Nucl.		Misincorporation								Total	
m^1A-1309	A1322	Sample:	G%	SD	A%	SD	T%	SD	C%.	SD	Avg	SD
		DLBCL	4.82	0.54	-	-	10.79	2.44	1.33	0.54	16.95	2.92
		RLN	3.86	0.23	-	-	8.91	0.16	1.75	0.11	14.52	0.48
		Cell lines	4.27	0.11	-	-	7.78	1.84	1.51	0.47	13.57	2.39
Modification	Nucl.											
m^3U-4500	U4530	Sample:	G%	SD	A%	SD	T%	SD	C%.	SD	Avg	SD
		DLBCL	5.74	1.27	23.88	1.95	-	-	0.29	0.11	29.92	3.11
		RLN	4.74	0.67	24.77	0.80	-	-	0.31	0.07	29.81	1.44
		Cell lines	5.71	0.65	27.60	1.62	-	-	0.33	0.04	33.63	2.26

*Numbering according to the snoRNA-LMBE-db, Nucl.: nucleotide number in the reference sequence, SD: standard deviation, Avg: average.

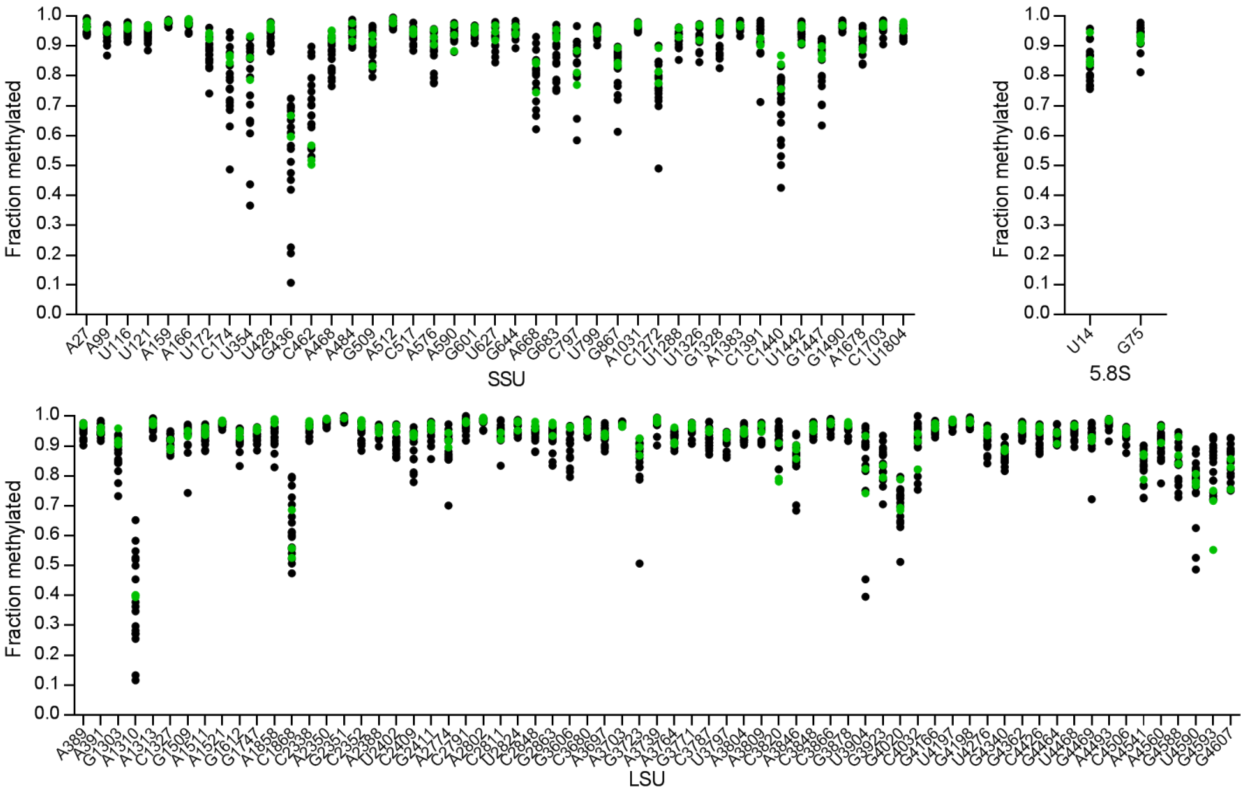

Figure S1. RiboMeth-seq profiles of tumors (black dots) and benign reactive lymph nodes (green dots). The graph shows the fraction of rRNA molecules methylated at the ribose at all known methylation sites. Data points are average values of triplicates. For graphical clarity standard deviations are not included but can be found together with the read-ends recorded from sequencing libraries that were used to calculate the values in **Supplementary Data S1**.

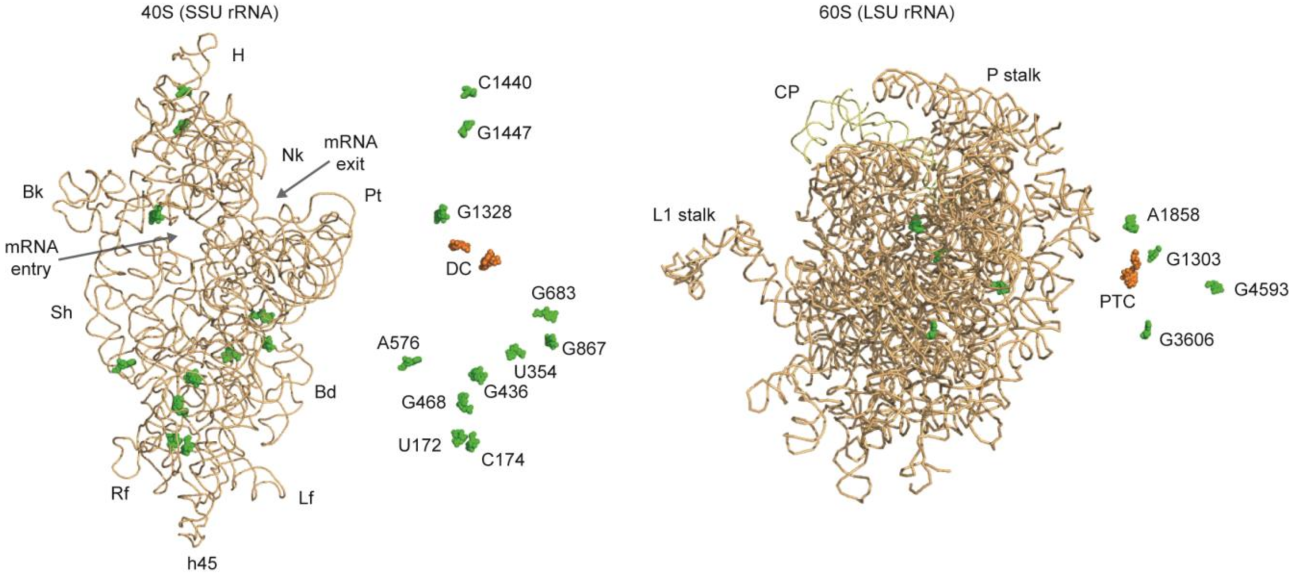

Figure S2. Depiction of the most affected 2’-O-Me sites in DLBCL on 3D models of the two human ribosomal subunits as viewed from the intersubunit face. SSU (left) and LSU (right) rRNA are in pale orange, 5S rRNA in yellow, and 2’-O-Me sites labeled as green spheres with a numbering key to the right of the structures. The sites were also described in **Table 1**. The drawings were based on PDB entry 4UG0. H: Head, Nk: Neck, Pt: Platform, Bd: Body, Lf: Left foot, Rf:

Right foot, Sh: Shoulder, Bk: Beak, CP: Central protuberance, DC: Decoding center (based on nucleotides G626, A1824, and A1825), and PTC: Peptidyl transferase center (based on nucleotides C3888, A4367, and U4501). Nucleotide numbering followed the same numbering system as with methylated nucleotides and labelled as orange spheres.

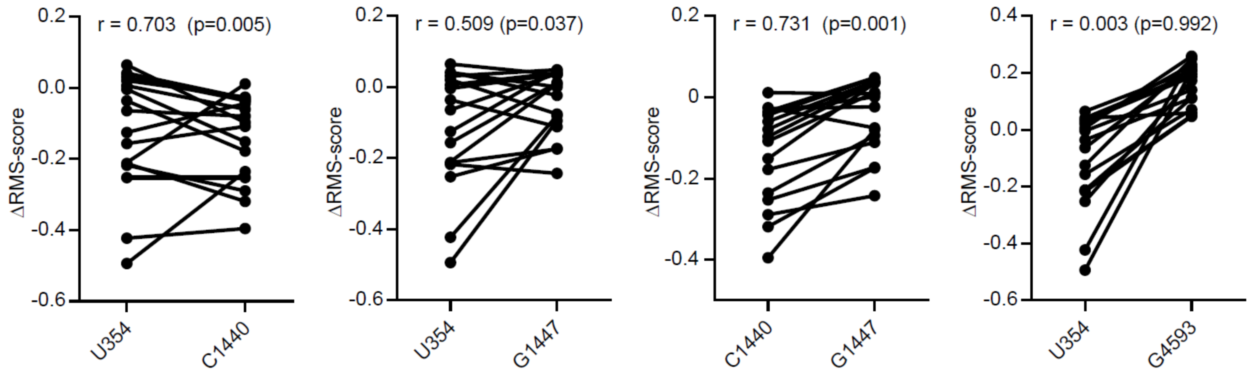

Figure S3. Analyses of co-varying methylation changes in DLBCL tumors. The graphs show pair-wise comparisons at selected sites in individual tumors using Spearman's rank correlations. The ΔRMS-scores were taken from **Figure 2A.**

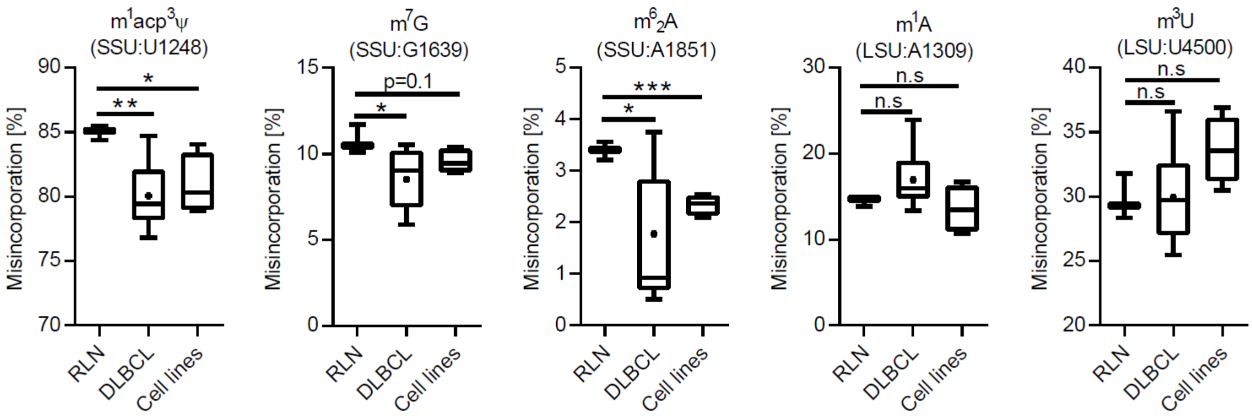

Figure S4. Box plots showing misincorporation in cDNA synthesis at base modifications on the WC-face of the nucleotide. The detailed data are shown in **Supplementary Table S3** and are extracted from a SNP-file (Single Nucleotide Polymorphism) that is part of the RiboMeth-seq analysis pipeline and used to reveal sequence variants in rRNA and sequencing errors due to homopolymeric stretches. Many nucleotide modifications, in particular at the WC-face of nucleotides, induce misincorporation during reverse transcription and these can be used as a proxy for modifications levels.

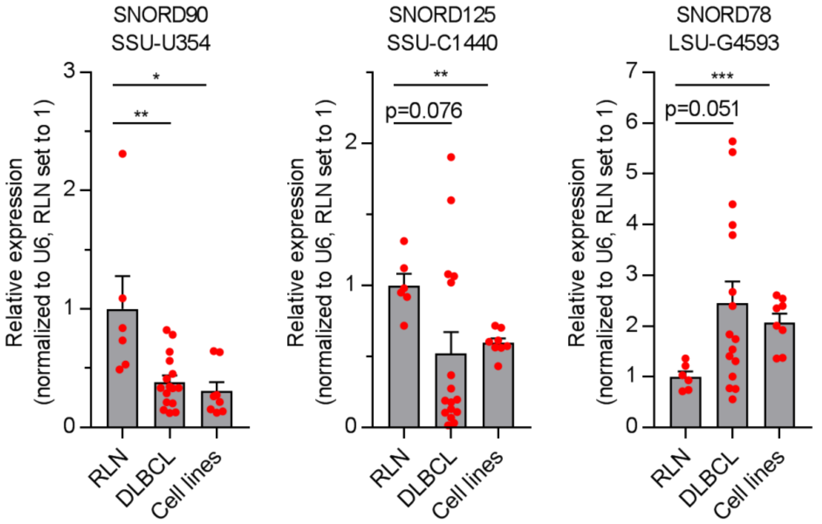

Figure S5. RT-qPCR analysis of SNORD90, -125, and -78 discussed in the main text. Whole cell RNA from 6 RLNs, 16 DLBCLs (**Supplementary Table S1**), and biological duplicates for each of the four cell lines (RL, HT, OCI-Ly3, and U-2932) were used for RT-qPCR and normalized against U6 snRNA. Primers are listed in **Supplementary Table S2**.

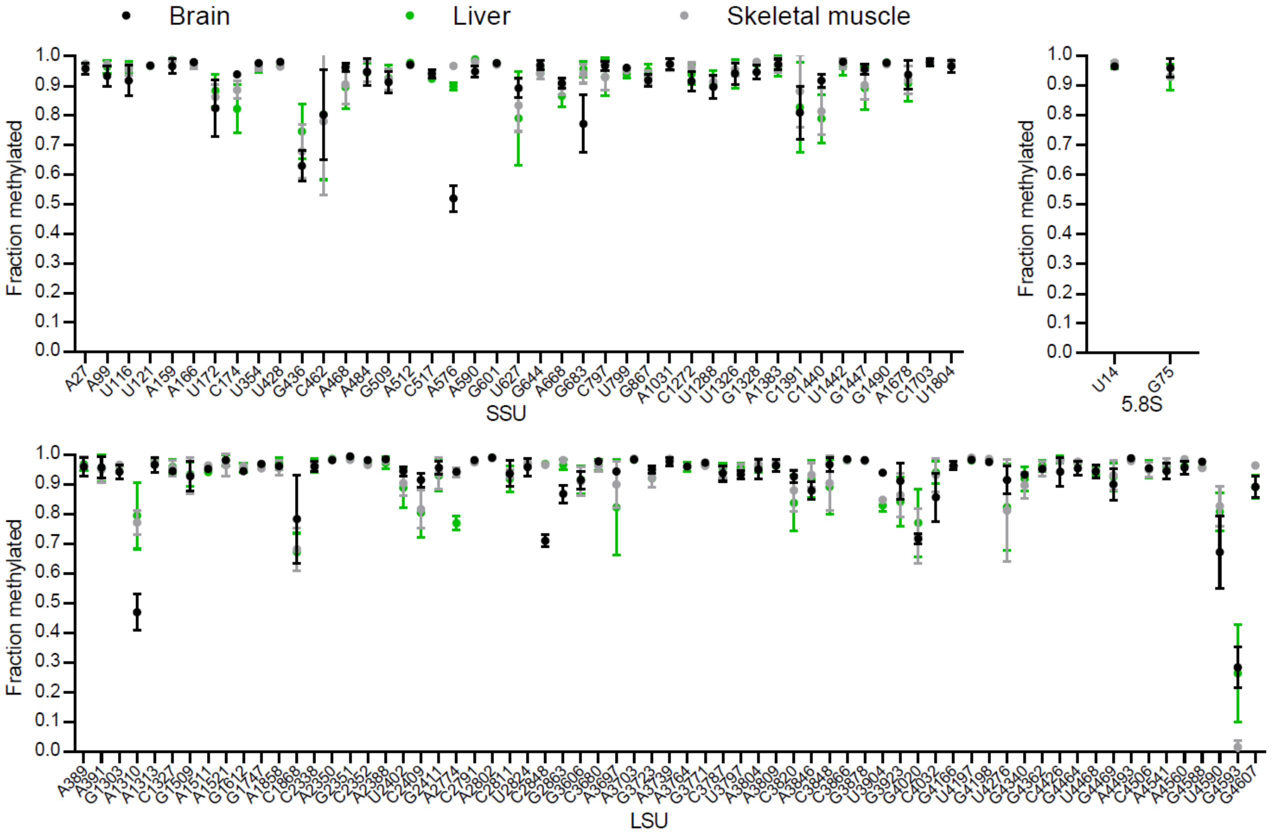

Figure S6. RiboMeth-seq profiles of three human tissues. The graph shows the fraction of rRNA molecules methylated at the ribose at all known methylation sites. The samples were each pooled from five donors and the analyses conducted in technical triplicates.
